# Heatstroke: a multicenter study in Southwestern China

**DOI:** 10.3389/fpubh.2024.1349753

**Published:** 2024-04-17

**Authors:** Lvyuan Shi, Bo Wang, Qin Wu, Jing Yang, Lietao Wang, Dingyuan Wan, Yucong Wang, Zhongxue Feng, Wei Zhang, Li Li, Wenhu Wang, Jun Chen, Xiaohua Ai, Jianwei Zheng, Zhongwei Zhang, Min He, Heat Stroke Research Group in Southwestern China

**Affiliations:** ^1^Department of Critical Care Medicine, West China Hospital, Sichuan University, Chengdu, Sichuan, China; ^2^Department of State Key Laboratory of Biotherapy and Cancer Center, West China Hospital, Sichuan University and Collaborative Innovation Center of Biotherapy, Chengdu, China; ^3^Department of Critical Care Medicine, The Second People's Hospital of Neijiang City, Neijiang, Sichuan, China; ^4^Department of Critical Care Medicine, Zizhong County People's Hospital, Neijiang, Sichuan, China; ^5^Department of Critical Care Medicine, The People's Hospital of Jianyang City, Jianyang, Sichuan, China; ^6^Department of Critical Care Medicine, The People's Hospital of Zhongjiang, Deyang, Sichuan, China; ^7^Department of Critical Care Medicine, Hejiang People's Hospital, Luzhou, Sichuan, China

**Keywords:** heatstroke, retrospective, intensive care units, multicenter, descriptive study, epidemiological patterns, clinical characteristics, patient outcomes

## Abstract

**Background:**

An increase in Heatstroke cases occurred in southwest China in 2022 due to factors like global warming, abnormal temperature rise, insufficient power supply, and other contributing factors. This resulted in a notable rise in Heatstroke patients experiencing varying degrees of organ dysfunction. This descriptive study aims to analyze the epidemiology and clinical outcomes of Heatstroke patients in the ICU, providing support for standardized diagnosis and treatment, ultimately enhancing the prognosis of Heatstroke.

**Methods:**

A retrospective, multicenter, descriptive analysis was conducted on Heatstroke patients admitted to ICUs across 83 hospitals in southwest China. Electronic medical records were utilized for data collection, encompassing various aspects such as epidemiological factors, onset symptoms, complications, laboratory data, concurrent infections, treatments, and patient outcomes.

**Results:**

The dataset primarily comprised classic heatstroke, with 477 males (55% of total). The patient population had a median age of 72 years (range: 63–80 years). The most common initial symptoms were fever, mental or behavioral abnormalities, and fainting. ICU treatment involved respiratory support, antibiotics, sedatives, and other interventions. Among the 700 ICU admissions, 213 patients had no infection, while 487 were diagnosed with infection, predominantly lower respiratory tract infection. Patients presenting with neurological symptoms initially (*n* = 715) exhibited higher ICU mortality risk compared to those without neurological symptoms (*n* = 104), with an odds ratio of 2.382 (95% CI 1.665, 4.870) (*p* = 0.017).

**Conclusion:**

In 2022, the majority of Heatstroke patients in southwest China experienced classical Heatstroke, with many acquiring infections upon admission to the ICU. Moreover, Heatstroke can result in diverse complications.

## Background

Heatstroke is a severe form of heat-related illness characterized by a rapid elevation of core body temperature exceeding 40°C, accompanied by central nervous system dysfunction (1, 2). Heatstroke can be classified into two main types: classical heatstroke and exertional heatstroke (1, 3). Classical heatstroke is typically associated with passive exposure to high temperature and humidity and often manifests in epidemic outbreaks. It primarily affects older people with pre-existing medical conditions. On the other hand, exertional heatstroke predominantly occurs in young, otherwise healthy individuals engaged in vigorous physical activities in hot or mildly hot environments (4). Within the intensive care unit (ICU) setting, the mortality rates for patients with exertional and classical heatstroke are reported as 26.5 and 63.2%, respectively (5).

Heatstroke can result in varying degrees of organ dysfunction (6). When the body’s core temperature surpasses 40°C and the compensatory mechanisms fail, cellular damage occurs (7). This, in turn, can trigger ischemia–reperfusion injury, excessive inflammatory response, and disseminated intravascular coagulation, leading to further impairment of organ function (8–10). The primary manifestations of heatstroke-induced organ dysfunction encompass central nervous system disorders, coagulation abnormalities (11), liver dysfunction (12, 13), circulatory failure, respiratory failure, and renal failure (14), among others (5, 15).

Considering the limited availability of multicenter descriptive data regarding intensive care unit (ICU) patients, this multicenter study was designed with the aim of describing and analyzing the characteristics of such patients. This effort aims to offer assistance in standardizing the diagnosis and treatment of Heatstroke, with the ultimate goal of enhancing the prognosis for Heatstroke patients.

## Materials and methods

### Study design and participants

This retrospective, multicenter, descriptive study was conducted to gather data on heatstroke patients admitted to intensive care units (ICUs) in southwest China from July 2022 to September 2022. A total of 83 units participated in this study. The inclusion criteria required patients to be admitted to the ICU with a diagnosis of heatstroke based on ICD coding. We instructed the specific personnel of each center to fill in the case report form (CRF) and record the indicators of the patients. The main observation index was the in-hospital mortality of patients with Heatstroke. The secondary observation index were the intervention measures and disease change records of the patients with Heatstroke during the treatment period, including different intervention measures and vital signs, respiratory support, nutritional status, incidence of complications, length of stay and so on. Ultimately, a total of 873 patients with heatstroke were included in the analysis.

### Data collection

Baseline characteristics, comorbidities, laboratory test results, treatment details, complications, and outcome indicators were collected through the completion of electronic medical record forms. Laboratory variables were derived from the initial examination of heatstroke patients upon their admission to the ICU.

### Statistical analysis

Continuous variables were presented as medians with interquartile ranges (IQRs), while categorical data were expressed as numbers and percentages. To compare continuous variables between groups, Student’s t-test or the Mann–Whitney U-test was employed, whereas the χ2 test was used for categorical variables. Fan charts were utilized to represent sex, type of heatstroke, type of infection, discharge from ICU, and discharge outcomes. Violin charts were employed to depict laboratory data upon ICU admission. Bar charts were used to display initial symptoms, complications, treatment, and outcomes. Cox regression analysis was conducted to assess the ICU outcome of patients with and without neurological symptoms, and subgroup analysis was performed based on factors such as gender, type of heatstroke, hypertension, diabetes, COPD, among others. A *p*-value of less than 0.05 was considered statistically significant. All statistical analyses were carried out using SPSS (version 25.0).

### Ethics declarations

The study was approved by the Ethics Committee of the West China Hospital of Sichuan University (IRB# 2022–1,542; Title: Exploration of clinical characteristics and severe risk factors of Heatstroke: a national multicenter study; The IRB approval date: October 13, 2022). All participants provided written informed consent before the study. The study was conducted in accordance with the principles of the Declaration of Helsinki. To ensure participant confidentiality, all data collected were coded and analyzed anonymously. In addition, the clinical registration number of the study is ChiCTR2200066314.

### Results

**Figure 1:**
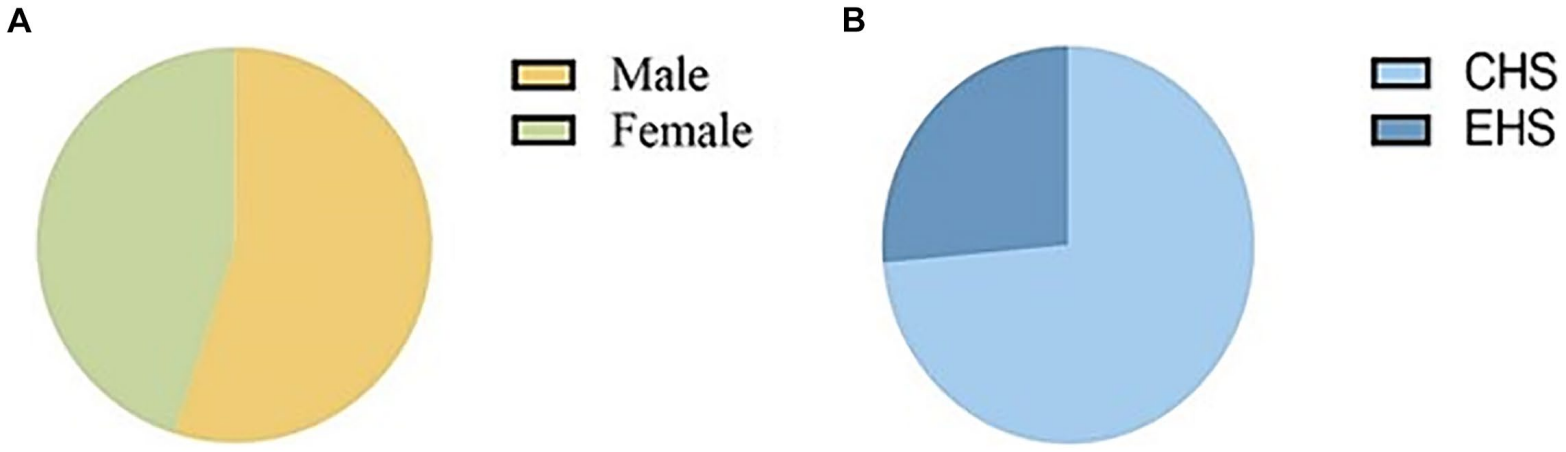
Pie chart of sex and typing of patients with heatstroke.

**Figure 2:**
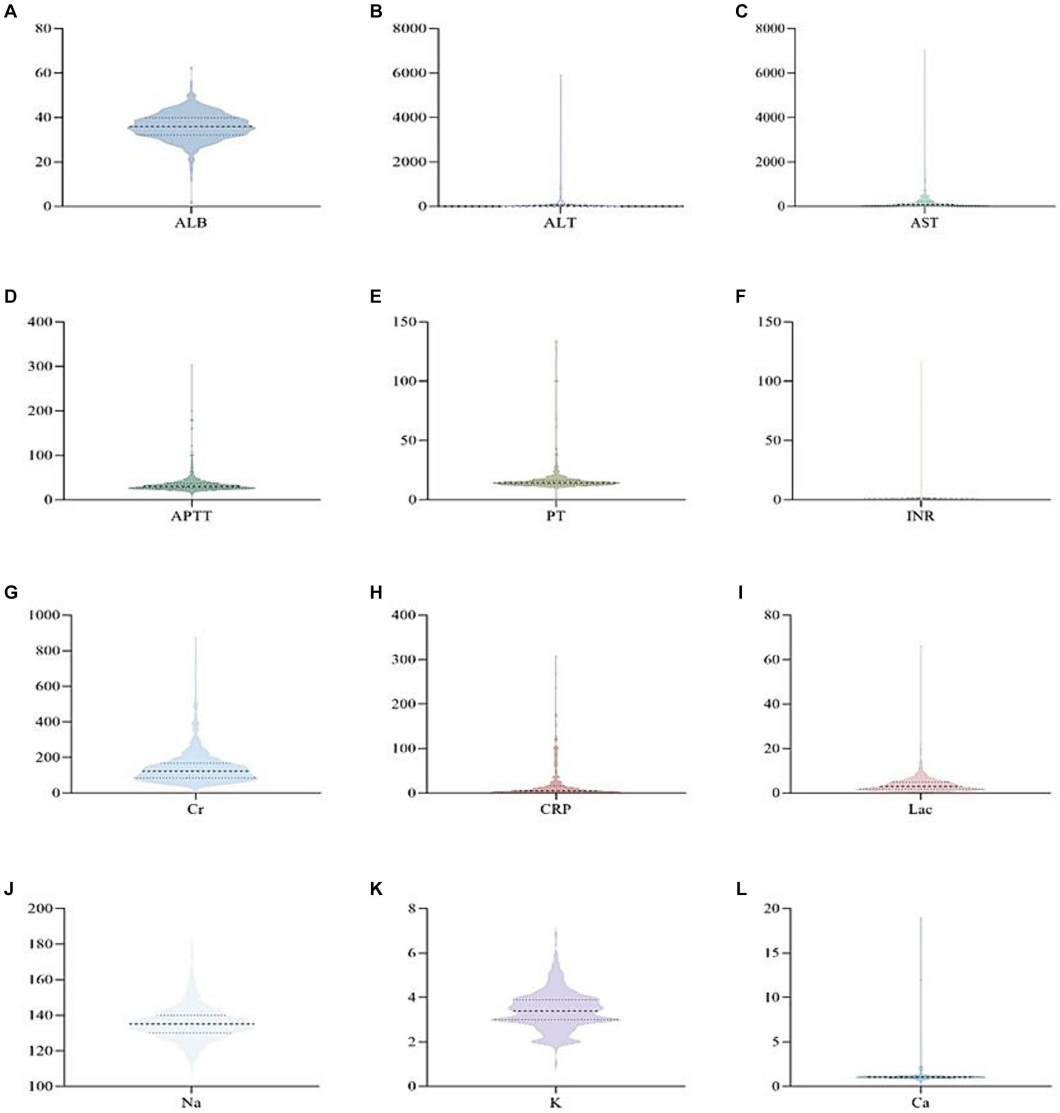
Violin pictures of the first laboratory results of patients with heat radiation disease when they were admitted to ICU.

Out of the 873 patients included in the study, 477 (55%) were male, and the majority, 565 (73.57%), were classified as having classical heat stroke (Figure 1). The median age of the patients was 72 years (IQR: 63–80), with a median body weight of 60 kg (IQR: 50–65), median height of 162 cm (IQR: 155–170), and median BMI of 22.05 (IQR: 20.41–24.22). The median temperature recorded during the initial measurement was 40.8°C (IQR: 40–41.4), and the median Glasgow Coma Scale (GCS) score was 6 (IQR: 4–10). The median length of stay in the ICU was 2 days (IQR: 1–4), and the median length of hospital stay was 5 days (IQR: 2–10). The ICU mortality rate was 19.6%, and the discharge mortality rate was 32.4% (Supplementary Table S1). Upon admission, the patients exhibited certain laboratory values. The serum albumin (ALB) level was 35.90 g/L (IQR: 32.13–39.80), alanine aminotransferase (ALT) level was 42.00 IU/L (IQR: 22.00–95.13), aspartate aminotransferase (AST) level was 85.50 IU/L (IQR: 41.00–216.90), activated partial thromboplastin time (APTT) level was 30.10 s (IQR: 25.60–36.40), prothrombin time (PT) level was 14.40 s (IQR: 12.98–16.45), international normalized ratio (INR) level was 1.22 (IQR: 1.10–1.41), serum creatinine (Cr) level was 122 umol/L (IQR: 83.55–168.53), C-reactive protein (CRP) level was 5 mg/L (IQR: 1.35–16.03), lactate (Lac) level was 3.00 mmol/L (IQR: 1.70–4.90), sodium (Na) level was 135.00 mmol/L (IQR: 130.00–140.00), potassium (K) level was 3.40 mmol/L (IQR: 3.00–3.90), and calcium (Ca) level was 1.04 mmol/L (IQR: 0.96–1.12) (Figure 2).

**Figure 3:**
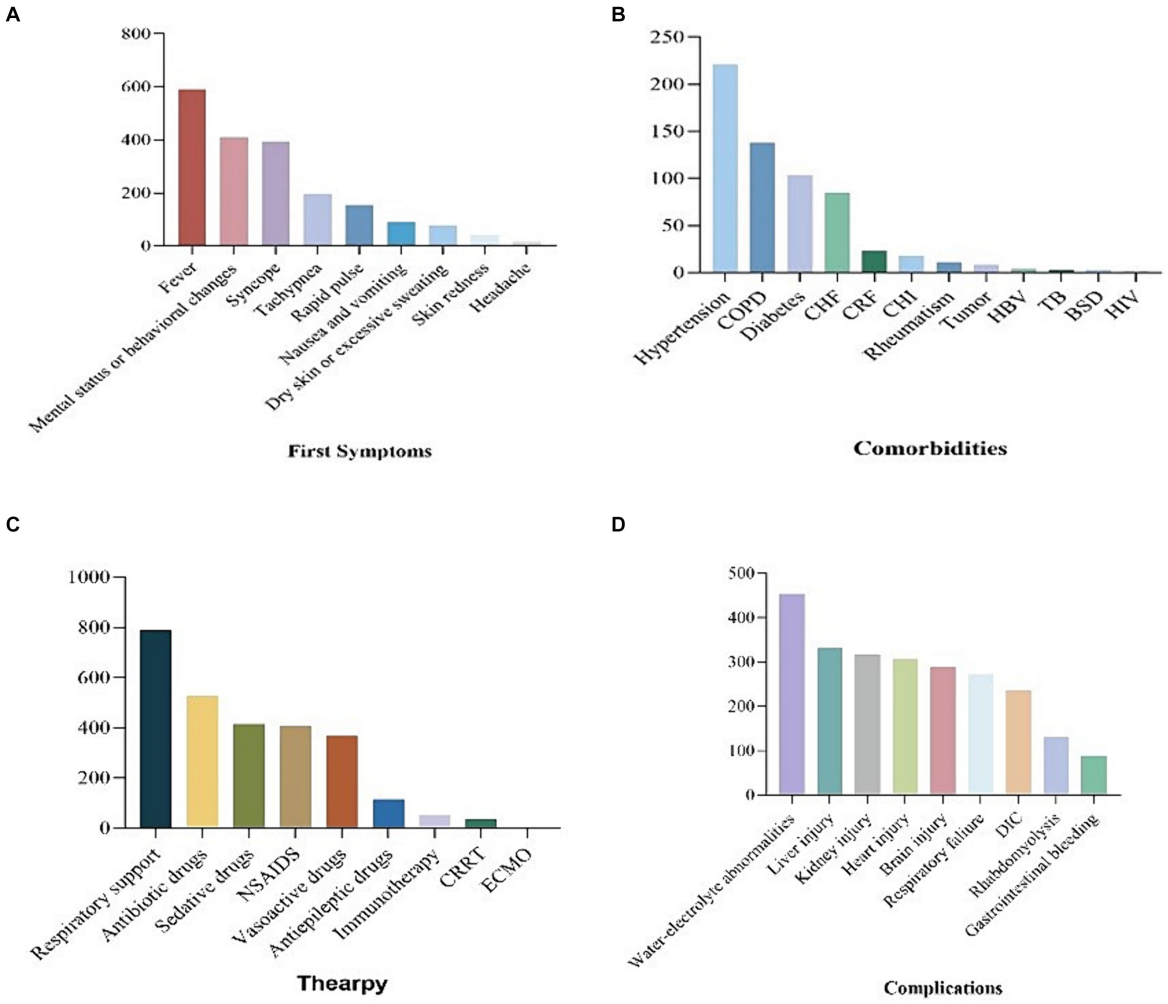
Bar chart of first symptom, comorbities, therapy and complications of patients with heatstroke.

The predominant initial symptoms observed in the majority of patients were fever (*n* = 591), followed by mental or behavioral abnormalities (*n* = 408), fainting (*n* = 392), shortness of breath (*n* = 195), tachycardia (*n* = 154), nausea and vomiting (*n* = 91), dry skin or excessive sweating (*n* = 76), skin redness (*n* = 41), headache (*n* = 16), and other symptoms. Among the Heatstroke patients admitted to the ICU, a significant proportion experienced various types of complications. Among the 474 patients for whom data was available, 200 (42.2%) had hypertension, 127 (26.8%) had chronic obstructive pulmonary disease (COPD), and 92 (19.4%) had diabetes. Additionally, other conditions were identified, including chronic cardiac insufficiency (*n* = 74), chronic liver insufficiency (*n* = 16), rheumatic immune disease (*n* = 11), tumor (*n* = 8), hepatitis B (*n* = 4), pulmonary tuberculosis (*n* = 3), hematological diseases (*n* = 3), and HIV (*n* = 1). Please refer to Figure 3 for a visual representation of these findings (Figure 3).

Patients with heatstroke in the ICU received various treatments, including respiratory support (*n* = 789), antibiotics (*n* = 526), sedatives (*n* = 415), non-steroidal anti-inflammatory drugs (NSAIDs) (*n* = 407), vasopressin (*n* = 367), antiepileptic drugs (*n* = 115), immunotherapy (*n* = 50), continuous renal replacement therapy (CRRT) (*n* = 34), and extracorporeal membrane oxygenation (ECMO) (*n* = 1). Among the patients who were discharged from the ICU, 364 survived, 148 died, 207 were transferred to general wards, 22 were transferred to other hospitals, and 15 were transferred to superior hospitals. Heatstroke patients in the ICU experienced various complications, including water and electrolyte disturbance (*n* = 454), liver function damage (*n* = 331), renal function damage (*n* = 316), myocardial damage (*n* = 306), central nervous system damage (*n* = 289), respiratory failure (*n* = 272), coagulation dysfunction (*n* = 235), rhabdomyolysis (*n* = 130), gastrointestinal bleeding (*n* = 89), and other complications (Figure 3).

**Figure 4:**
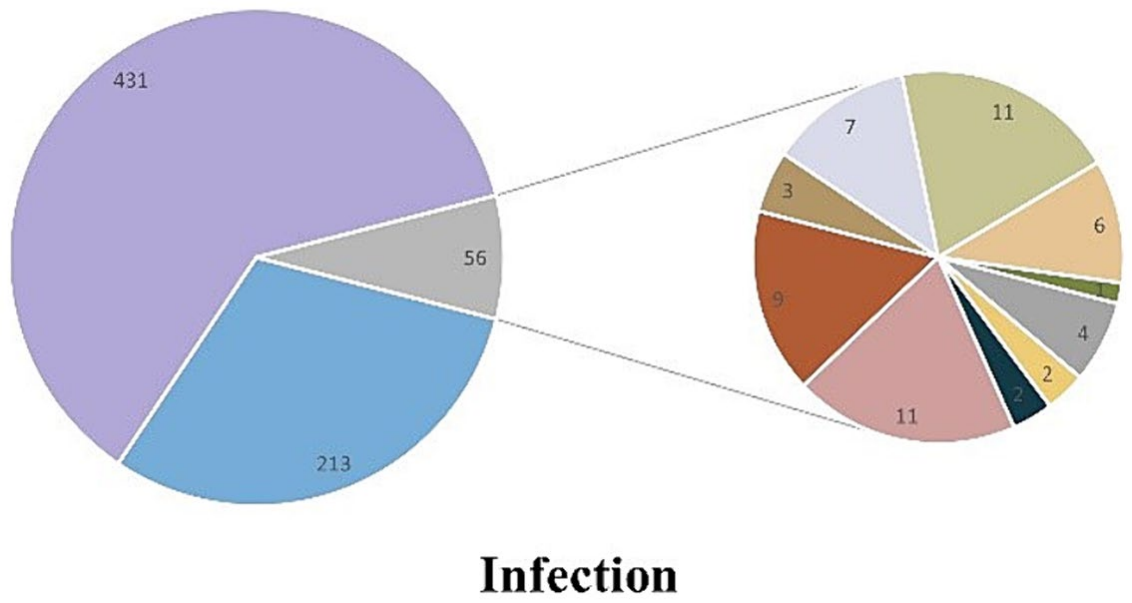
Compound pie chart of infection in patients with heatstroke.

Among the 700 patients who were initially admitted to the ICU, 213 patients did not have any infections, while 487 patients were infected. The main types of infection observed were simple lower respiratory tract infection (*n* = 431), bloodstream infection (*n* = 4), urinary tract infection (*n* = 2), abdominal infection (*n* = 2), lower respiratory tract infection with bloodstream infection (*n* = 11), lower respiratory tract infection with urinary tract infection (*n* = 9), lower respiratory tract infection with abdominal infection (*n* = 3), lower respiratory tract infection with skin and soft tissue infection (*n* = 7), and other uncertain types of infection (Figure 4).

The patients were categorized into two groups based on their first episode symptoms: patients with neurological symptoms as the first episode (*n* = 715) and patients without neurological symptoms as the first episode (*n* = 104). For patients with neurological symptoms as the first episode, the discharge mortality rate was 34.7% and the ICU mortality was 20.7%. Patients without neurological symptoms as the first episode had a discharge mortality rate of 15.5% and a ICU mortality of 9.4%. It was observed that the risk of ICU death was higher in patients with neurological symptoms as the first episode, with a hazard ratio of 2.382 (95% CI 1.665, 4.870) and a *p*-value of 0.017. Subgroup analysis was conducted considering factors such as sex, type of heatstroke, hypertension, diabetes, and COPD. However, no significant difference in ICU outcome was found between patients with and without neurological symptoms (Supplementary Table S2).

## Discussions

“Heatstroke” represents the most critical stage in the progression of heat-related illnesses. It occurs when there is an excessive buildup of heat in the body, surpassing its capacity to dissipate heat, typically following intense physical exertion or exposure to extreme thermal conditions (16). Clinical manifestations of heatstroke include central nervous system (CNS) dysfunction, multiple organ failure, and a significant rise in body temperature, often exceeding 40.0°C (5, 17). In our dataset, the median of the initial temperature measurement among heatstroke patients was recorded as 40.8 (40, 41.4) °C. Heatstroke can be further classified into classical and exertional types based on its underlying causes (1, 2). Among our study participants, 565 (73.57%) patients were identified as having classical heatstroke. Classical heatstroke primarily occurs when individuals are exposed to high ambient temperatures and experience impaired heat dissipation mechanisms. This type of heatstroke is commonly observed in older adults, children, and individuals with underlying health conditions (18). In line with this observation, the median age of patients with classical heatstroke in our dataset was 75 (66, 82) in Supplementary Table S1, reinforcing the aforementioned conclusion.

Cardiovascular disease, respiratory disease, and diabetes are known to impair the body’s ability to adapt to environmental changes (16). Among the 474 Heatstroke patients in our study, 200 (42.2%) had hypertension, 127 (26.8%) had chronic obstructive pulmonary disease, and 92 (19.4%) had diabetes. Hypertension is characterized by increased peripheral vascular resistance and various alterations in peripheral circulation, including vascular smooth muscle hypertrophy and vascular remodeling. These changes can impair the regulation of skin blood flow, thus compromising the body’s ability to regulate core temperature (19–21). Epidemiological evidence suggests that individuals with respiratory conditions such as asthma, chronic obstructive pulmonary disease, lung cancer, influenza, pneumonia, bronchitis, tuberculosis, and cystic fibrosis may be more susceptible to the effects of heat exposure (22, 23). A meta-analysis has reported that people with respiratory diseases have a higher risk of mortality during heat waves (odds ratio 1.61, 95% confidence interval 1.2 to 2.1) (24). Additionally, studies have shown that poor glycemic control and the presence of neuropathy in individuals with type 2 diabetes can affect the sweating response, further impacting heat regulation (25, 26). However, the specific mechanisms underlying the relationship between these factors and heatstroke are not yet fully understood, and further research is needed to elucidate their interplay (27, 28).

Heatstroke can result in various degrees of organ dysfunction (29). The inflammatory response observed in heatstroke bears similarities to systemic inflammatory response syndrome (SIRS), which can rapidly deteriorate clinical status and lead to disseminated intravascular coagulation, multiple organ failure syndrome, and ultimately, death (17). Among heatstroke patients in the ICU, there are diverse complications, including disturbances in water and electrolyte balance (*n* = 454), liver function impairment (*n* = 331), renal function impairment (*n* = 316), myocardial damage (*n* = 306), central nervous system damage (*n* = 289), respiratory failure (*n* = 272), coagulation dysfunction (*n* = 235), rhabdomyolysis (*n* = 130), gastrointestinal bleeding (*n* = 89), and others. Retrospective studies have identified metabolic acidosis as a prominent acid–base alteration (30). In heatstroke-induced multiple organ dysfunction syndrome (MODS), the liver is recognized as one of the organs most susceptible to early damage (31). The mechanism underlying liver injury in heatstroke involves not only systemic factors such as systemic inflammatory reactions and coagulation dysfunction but also pathological mechanisms like aberrant hepatocyte death and impaired function of Kupffer cells (32).

Neurological alterations are significant features observed in both classical heatstroke and exertional heatstroke (5). These changes typically manifest suddenly in approximately two-thirds of patients and can present as mental disorders, including confusion, delirium, or drowsiness (33–35). The patient cohort was categorized into two groups: those with neurological symptoms as the initial episode (*n* = 715) and those without neurological symptoms as the initial episode (*n* = 104). It was determined that patients with neurological symptoms as the first episode had a higher risk of ICU mortality, with a hazard ratio of 2.382 (95% CI 1.665, 4.870) (*p* = 0.017). The susceptibility of the central nervous system to hyperthermia leads to increased metabolic rate in the brain, reduced blood flow, enhanced permeability of the blood–brain barrier, and greater infiltration of inflammatory factors and pathogens into the brain (36). Consequently, the central nervous system represents a primary target organ in severe heatstroke (37). Previous clinical investigations have demonstrated that neurological disabilities exert an adverse impact on long-term ICU mortality in heat-related diseases (38–40).

Currently, treatment for heat radiation disease remains supportive, lacking a specific therapy (5). This condition arises when the body’s heat accumulation surpasses its capacity to dissipate heat, either due to inadequate thermoregulation in an uncompensated thermal environment or a breakdown of thermoregulation caused by dehydration (41, 42). Given the era of global warming, adopting effective preventive measures against heat radiation disease appears crucial.

## Limitations

This study has several limitations that should be acknowledged. Firstly, the geographical scope of the study was predominantly limited to the southwest region of China. To enhance the generalizability of the findings, it is imperative to include more diverse regions in future research. Secondly, being a retrospective multicenter cohort study, inherent biases in data collection are inevitable. For example, information bias may occur, including systematic errors in the collection and collation of relevant disease data. It is important to recognize that the study findings may be influenced by these biases. Thirdly, the relatively small sample size of patients with Exertional Heatstroke (EHS) in our study might diminish the statistical power and precision of the analysis. Nevertheless, no comprehensive investigation has been conducted to determine the potential neuroprotective effects of induced hypothermia on patients with Heatstroke, nor its impact on prognosis. Consequently, our research team intends to initiate a multicenter prospective study in 2023, aimed at addressing this knowledge gap and resolving this issue.

## Conclusion

Heatstroke can result in varying degrees of organ dysfunction. Furthermore, the intensive care unit (ICU) outcomes for patients with heatstroke presenting with initial symptoms related to the nervous system are poorer compared to those without such symptoms. In 2022, most of the patients with Heatstroke in southwest China are patients with classical Heatstroke. Most of the patients were infected when they were admitted to ICU. In addition, Heatstroke can lead to different types of complications. Due to the large geographical span of China, the occurrence of Heatstroke is different in different regions. In the future, we plan to include as many centers and more cases as possible in different regions, so as to provide more abundant evidence for Heatstroke.

## Data availability statement

The raw data supporting the conclusions of this article will be made available by the authors, without undue reservation.

## Ethics statement

The studies involving humans were approved by Ethical approval was obtained from the ethics committee of the West China Hospital of Sichuan University (NO: 2022-1542). The studies were conducted in accordance with the local legislation and institutional requirements. The participants provided their written informed consent to participate in this study.

## Author contributions

LS: Writing – original draft. BW: Writing – original draft. QW: Writing – review & editing. JY: Methodology, Writing – original draft. LW: Writing – original draft. DW: Writing – original draft. YW: Writing – original draft. ZF: Writing – review & editing. WZ: Writing – review & editing. LL: Writing – review & editing. WW: Writing – review & editing. JC: Writing – review & editing. XA: Writing – review & editing. JZ: Writing – review & editing. ZZ: Supervision, Writing – review & editing. MH: Writing – review & editing.

## Glossary

ICU: intensive care unit
CHS: Classical Heatstroke
EHS: Exertional Heatstroke
IQRs: interquartile ranges
COPD: chronic obstructive pulmonary disease
NSAIDs: non-steroidal anti-inflammatory drugs
CRRT: continuous renal replacement therapy
ECMO: extracorporeal membrane oxygenation
CNS: central nervous system
SIRS: systemic inflammatory response syndrome
CHF: Chronic Heart Failure
CRF: Chronic Renal Failure
CHI: Chronic Hepatic Insufficiency
HBV: Hepatitis B
TB: Tuberculosis
BSD: Blood System Disease
HIV: Human Immune Deficiency virus
DIC: Disseminated Intravascular Coagulation
NS: patients with neurological symptoms as the first episode
non-NS: patients without neurological symptoms as the first episode
NS: neurological symptoms as the first episode
non-NS: non-neurological symptoms as the first episode
GCS: Glasgow Coma Scale
ALB: albumin
ALT: alanine aminotransferase
AST: aspartate aminotransferase
APTT: activated partial thromboplastin time
PT: prothrombin time
INR: international normalized ratio
BNP: brain natriuretic peptide
CKMB: creatine kinase isoenzymes
CK: creatine kinase
BUN: blood urea nitrogen
Cr: serum creatinine
CysC: CystatinC
IL-6: interleukin 6
CRP: C-reactive protein
PCT: procalcitonin
Lac: lactate
Na: sodium ion
K: potassium ion
Ca: calcium ion
WBC: white blood cell
PLT: platelet
