# Supplementary material for: Heatstroke: a multicenter study in Southwestern China
Source: Front Public Health. 2024 Apr 17;12:1349753. doi: 10.3389/fpubh.2024.1349753 (PMC11064700; doi:10.3389/fpubh.2024.1349753)

Table S1: Demographic and baseline characteristics of patients with Heatstroke

|  | **Total=873** | | **Type(Total=768)** | | **CHS(Total=565)** | | **EHS(Total=203)** | |  |
| --- | --- | --- | --- | --- | --- | --- | --- | --- | --- |
| **Parameter** | Miss | **N** | Miss | **N** | Miss | **CHS(Total=565)** | Miss | **N** | p-value |
| Male(%) | 5 | 477(55%) | 0 | 430(56%) | 0 | 285（50.4%） | 0 | 145（71.4%） | 0.000 |
| Age(years) | 5 | 72（63，80） | 0 | 72（63，80） | 0 | 75（66，82） | 0 | 64（56，73） | 0.000 |
| Weight(kg) | 5 | 60（50，65） | 0 | 60(50,65.) | 0 | 60(50,65) | 0 | 60(56,70) | 0.000 |
| Height(cm) | 5 | 162（155，170） | 0 | 162(155.0,170.0) | 0 | 160(155,168) | 0 | 165(160,170) | 0.000 |
| BMI | 5 | 22.05(20.41,24.22) | 0 | 22.05(20.40,24.22) | 0 | 22.03(20.12,24.03) | 0 | 22.86(20.76,24.38) | 0.002 |
| First temperature(℃) | 102 | 40.8(40,41.4) | 77 | 40.9(40.0,41.4) | 59 | 40.5(40,41.2) | 18 | 41(40,41.6) | 0.008 |
| GCS | 107 | 6(4,10) | 92 | 6(4,10) | 56 | 6(4,10) | 36 | 6(3,9) | 0.020 |
| **Laboratory results** | | | | | | | | | |
| ALB(g/L) | 209 | 35.90（32.13，39.80） | 176 | 35.9(32.3,39.9) | 115 | 35.9(32.1,39.6) | 61 | 36.25(32.88,41) | 0.324 |
| ALT(IU/L) | 203 | 42.00(22.00,95.13) | 174 | 41(21,94) | 115 | 37.55(19.08,91.25) | 59 | 45.75(27.25,116.63) | 0.008 |
| AST(IU/L) | 208 | 85.50(41.00,216.90) | 179 | 84(40,211) | 116 | 78(39.1,206.5) | 63 | 109(43.25,229.88) | 0.091 |
| APTT(s) | 233 | 30.10(25.60,36.40) | 202 | 29.45(25.6,36) | 140 | 29.2(25.6,34.5) | 62 | 30.1(25.6,38.05) | 0.213 |
| PT(s) | 236 | 14.40(12.98,16.45) | 205 | 14.4(12.9,16.6) | 141 | 14.4(13,16.4) | 64 | 14.5(12.4,16.9) | 0.656 |
| D-Dimer(mg/I FEU) | 314 | 4.08(1.68,12.36) | 271 | 4(1.69,11.97) | 191 | 4.21(1.86,12.05) | 80 | 3.6(1.3,12) | 0.203 |
| INR | 255 | 1.22(1.10,1.41) | 224 | 1.22(1.1,1.4) | 157 | 1.21(1.1,1.4) | 67 | 1.24(1.07,1.48) | 0.807 |
| BNP(ng/L) | 619 | 192.22(47.65,828,89) | 537 | 190.3(52.43,821) | 398 | 212(71,890) | 139 | 83.65(16.5,596.25) | 0.012 |
| CKMB(ng/ml) | 359 | 11.76(3.00,37.00) | 311 | 11(3,35.05) | 211 | 11.07(3,36) | 100 | 10(2.8,30.19) | 0.668 |
| Troponin(ng/L) | 532 | 4.62(0.14,198.45) | 473 | 5.21(0.16,197) | 333 | 7.60(0.17,300.5) | 140 | 4.60(0.15,100) | 0.141 |
| CK(mmol/L) | 621 | 470.50(182.25,2027.75) | 541 | 486(188,1776) | 395 | 455.95(187.43.1850) | 146 | 550(192.5,1587) | 0.825 |
| BUN(mmol/L) | 272 | 8.55(6.47,11.77) | 231 | 8.44(6.5,11.69) | 162 | 8.55(6.62,12.2) | 69 | 7.89(6.22,10.30) | 0.068 |
| Cr(umol/L) | 121 | 122(83.55,168.53) | 108 | 122(84.03,167.75) | 72 | 123(87,169) | 36 | 110(77.3,165.8) | 0.169 |
| CysC(mg/L) | 486 | 1.25(0.9,1.85) | 429 | 1.25(0.9,1.85) | 318 | 1.3(0.95,1.94) | 111 | 1.07(0.86,1.75) | 0.048 |
| IL-6(pg/ml) | 755 | 95.79(22.15,632.85) | 664 | 114.05(19.31,167.5) | 493 | 82.97(12.10,500.33) | 171 | 183.15(32.47,816,28) | 0.234 |
| CRP(mg/L) | 527 | 5(1.35,16.03) | 473 | 5(1.1,16.12) | 342 | 5(1,14.5) | 131 | 8.4(1.84,23.7) | 0.313 |
| PCT(ug/L) | 344 | 3,64(0.52,15.02) | 297 | 3.25(0.49,14.57) | 205 | 4.25(0.69,18.12) | 92 | 0.97(0.2,7.75) | 0.000 |
| Lac(mmol/L) | 178 | 3.00(1.70,4.90) | 151 | 3(1.7,5.0) | 101 | 2.95(1.7,4.9) | 50 | 3.1(1.7,5.05) | 0.545 |
| Na(mmol/L) | 191 | 135.00(130.00,140.00) | 162 | 135(130,140) | 110 | 134.3(129,140) | 52 | 136(131,141) | 0.110 |
| K(mmol/L) | 176 | 3.40(3.00,3.90) | 151 | 3.4(3,3.9) | 105 | 3.3(2.9,3.8) | 46 | 3.6(3,4) | 0.000 |
| Ca(mmol/L) | 274 | 1.04(0.96,1.12) | 237 | 1.04(0.96,1.12) | 171 | 1.03(0.96,1.1) | 66 | 1.07(1,1.14) | 0.003 |
| WBC(*10^9/L) | 161 | 11.30(8.30,15.50) | 135 | 11.3(8.25,15.5) | 90 | 11.4(8.7,15.5) | 45 | 10.4(7,14.43) | 0.002 |
| PLT(*10^9/L) | 106 | 102.00(64.00,152.00) | 93 | 103(64,156) | 63 | 107(67.75,162) | 30 | 95(55.5,139) | 0.012 |
| **Outcomes** | | | | | | | | | |
| ICU Mortality(%) | 117 | 148(19.6%) | 103 | 129(19.4%) | 71 | 95(19.2%) | 32 | 34(19.9%) | 0.853 |
| Discharge Mortality(%) | 172 | 227(32.4%) | 144 | 205(32.9%) | 101 | 157(33.8%) | 43 | 48(30.0%) | 0.373 |
| Days in ICU | 146 | 2(1,4) | 125 | 2(1,4) | 87 | 2(1,4) | 38 | 2(1,5) | 0.163 |
| Hospitalized days | 277 | 5(2,10) | 234 | 5(2,10) | 164 | 5(2,9) | 70 | 5(2,11) | 0.396 |

Table S2: A group analysis of the outcome of ICU between patients with heatstroke with neurological symptoms as the first episode and patients with non-neurological symptoms as the first episode


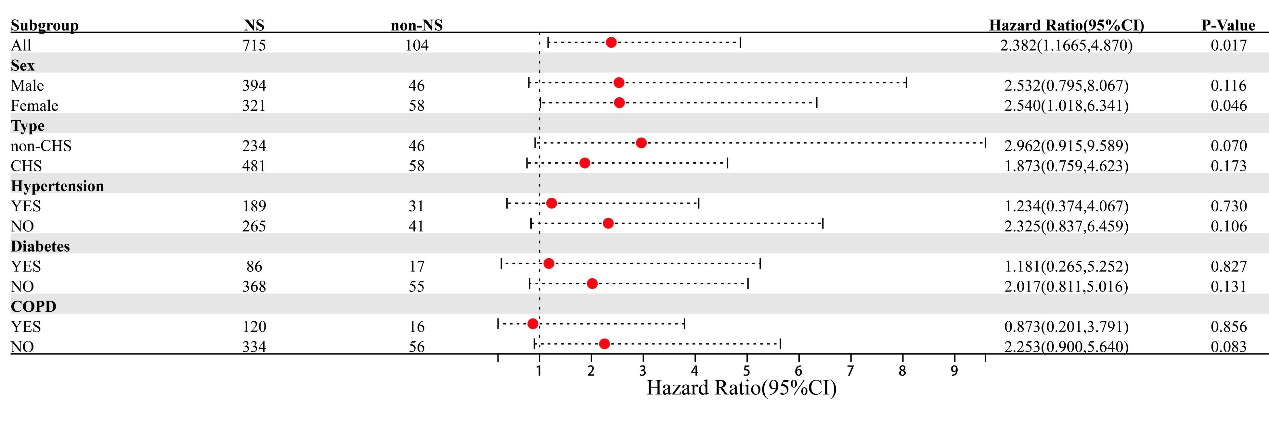

Supplement: Supplementary file 1 [file Table_1.DOCX]
